# In Vitro Antibacterial Activity of Crude Extracts of *Artocarpus heterophyllus* Seeds against Selected Diarrhoea-Causing Superbug Bacteria

**DOI:** 10.1155/2020/9813970

**Published:** 2020-09-07

**Authors:** Asio Eve, Adamu Almustapha Aliero, Doreen Nalubiri, Rasheed Omotayo Adeyemo, Saheed Adekunle Akinola, Theophilus Pius, Saphurah Nabaasa, Susan Nabukeera, Bashir Alkali, Ibrahim Ntulume

**Affiliations:** ^1^Department of Medical Laboratory Sciences, Kampala International University Western Campus, P.O Box 71 Bushenyi, Ishaka, Uganda; ^2^Department of Microbiology and Immunology, Faculty of Biomedical Sciences, Kampala International University Western Campus, P.O Box 71 Bushenyi, Ishaka, Uganda; ^3^School of Medicine, College of Health Sciences, Makerere University, P.O Box 7062, Kampala, Uganda; ^4^Department of Medical Laboratory Sciences, Kampala International University Teaching Hospital Western Campus, P.O Box 71 Bushenyi, Ishaka, Uganda

## Abstract

The current upsurge in resistance to conventional antibiotics, as well as high cost of orthodox medical treatment, called for the use of medicinal plants as an alternative therapy. This research was aimed at determining the antibacterial activity of *Artocarpus heterophyllus* seed extracts (Jackfruit as it is locally called) in the treatment of diarrhoea. Ethanolic and hexanolic seed crude extracts of the plant were screened for antidiarrhoeal activity against bacteria isolated from clinical samples (methicillin-resistant and susceptible *Staphylococcus aureus,* multidrug-resistant *Pseudomonas aeruginosa*, ciprofloxacin-resistant *Salmonella typhimurium*, and third-generation cephalosporin-resistant *Escherichia coli*). Plant phytochemical screening was conducted using standard methods. The antibacterial activity was carried out using the agar well diffusion method and compared to the standard antibiotics ceftriaxone and vancomycin. The minimum inhibitory concentration was determined by the microbroth dilution method, whereas the minimum bactericidal concentration was determined by plating out from microtitre plates with no visible growth. The results of phytochemical screening revealed the presence of tannins, flavonoids, reducing sugars, cardiac glycosides, saponins, and steroids from the prepared crude extracts. The ethanolic and hexanolic extracts had activity on multidrug-resistant *Pseudomonas aeruginosa,* methicillin-resistant *Staphylococcus aureus,* and methicillin-susceptible *Staphylococcus aureus* with the mean and standard error zone of inhibition that ranged from 8.5 ± 0.5 to 16.5 ± 0.25 mm; however, the extracts were found not to have activity on resistant *E. coli* and *Salmonella typhimurium*. The ethanolic crude extract had the lowest MIC and MBC values of 31.25 and 125 mg/ml, respectively, compared to the hexane extract which had the MIC and MBC values of 62.50 and 250 mg/ml, respectively. This provides the evidence for its usage as an alternative herbal remedy for the treatment of diarrhoea caused by susceptible and methicillin-resistant *Staphylococcus aureus* and multidrug resistant *Pseudomonas aeruginosa*.

## 1. Introduction

Diarrhoea is the passage of three or more abnomally/increased amount of loose liquid stools per day which deviates from an individual's usual pattern [1]. It is one of the main waterborne diseases considered to be endemic in many regions of the world and considered as a major health threat in both tropical and subtropical developing countries [2]. Furthermore, the gastrointestinal infections are the major cause of morbidity and mortality throughout the world and, particularly, in sub-Saharan Africa and South Asia, with high rates of 17.9% [3] or 50 to 150 per 100,000 individuals [4] that manifest infectious diarrhoea. Still, approximately 2.5 billion cases of diarrhoea occurred globally in 2013 which resulted in 1.5 billion death among children in South Asia and Africa [5]. According to a recent global burden of disease study (GBD) report, it is estimated that 1.6 million people died worldwide from diarrhoeal diseases [6]. In Uganda, approximately 230,000 Ugandans including 19,700 children less than five years of age die each year from diarrhoea with nearly 90% of which is directly attributed to poor water sanitation and hygiene [7]. Thus, diarrhoeal infection remains a second global leading cause of infant mortality after pneumonia with 17% prevalence [8].

The major causative agents of infectious diarrhoea in humans include the following: a wide variety of bacteria, viruses, and parasites [9]. Bacteria are well-known causative agents of gastrointestinal diseases which contribute to a majority of infectious diarrhoeal cases recorded worldwide [9]. The infectious bacteria include *Staphylococcus aureus*, *Streptococcus faecalis*, *Salmonella typhimurium*, *Pseudomonas aeruginosa*, and different groups of diarrhoeagenic *E. coli* [9]. These infectious agents associated with diarrhoea are transmitted chiefly through the faecal-oral route, with an estimated 94% disease burden attributable to the environment and associated with risk factors such as unsafe drinking water and poor sanitation and personal hygiene [7].

There are a number of signs and symptoms associated with infectious diarrhoea depending on the bacterial species and age of the patient, such as frequent loose (watery) stools, frequent abdominal cramps, bloating, abdominal pain and fever, bleeding from the rectum (back passage), blood in the stool, and light headache/dizziness from dehydration [10]. In terms of severity, the stools may be very watery for a prolonged period lasting longer than a week or it usually lasts two to four days [11]. There has been a rising prevalence of resistance in these causative bacteria to commonly used antimicrobials in the last 15 years [12]. The 2017World Health Organization (WHO) global priority list of pathogens ranked these diarrhoea-causing pathogens in the highest priority category (i.e., critical) that require development of novel antibiotics to combat their related infections [13]. According to the CDC's [14] report on antibiotic resistance, it is shown that over 2.5 million cases and over 35,000 deaths occurred each year due to antibiotic-resistant infections [14]. Moreover, a global antimicrobial resistance report by O'neill [15] showed that diarrhoeal diseases have caused over 1.4 million deaths every year attributable to antimicrobial resistance compared to other major causes of death. With the continued high attack rates, a desk study carried out by the Water and Sanitation Program (USP) has shown that the Ugandan government losses about US$ 177 million each year, an equivalent of US$ 5.5 per person in Uganda per year to combat waterborne-related diseases [16].

The acceptance of traditional medicine as an alternative form of health care and the development of microbial resistance to the available antibiotics have led researchers to investigate the antimicrobial activity of herbal plants [17]. Currently, the World Health Organization (WHO) estimates that 80% of the world's population use herbal medicine in treatment of several ailments [18], and this shift to herbal therapy can be attributed to the low cost and availability of herbal plants across the globe compared to conventional antibiotics. However, Vadhana et al. [19] reported the development of Herbal antimicrobial drug resistance in most pathogens which necessitates the need to explore other herbal plants and isolate potential phytochemicals that exhibit greater antibacterial effect.

Amongst plants with medicinal importance, *Artocarpus heterophyllus* is widely distributed throughout tropical Africa, and it is documented to exhibit several ethnomedicinal uses in Uganda. The plant parts of *A. heterophyllus* are currently used as traditional medicines for the treatment of asthma, wound healing, ulcers, dermatitis, and cough [20], while the seeds are used to cure diarrhoea, stomach ache, bowel and bladder disorders among others [21]. Despite the availability of many effective antibacterial drugs, most of them are relatively expensive in addition to their negative side effects. However, the existing studies conducted on *A. heterophyllus* seeds did not document its efficacy on antibiotic-resistant enteric bacteria thus, the current study necessitated to address this literature gap which will be a guide for future therapeutic application. Therefore, validating the antibacterial activity of *A. heterophyllus* could provide an alternative, accessible, and relatively cheap treatment of infectious diarrhoea caused by superbug bacteria [20]. Additionally, it is well known that the phytochemical composition of jackfruit varies considerably with the geographic origin and cultivation methods used and so is its antibacterial activities as reported by Senjobi et al. [22]. Hence, the present study was conducted to evaluate the in vitro antibacterial activities of Ugandan local jackfruit (*A. heterophyllus*) seeds against selected diarrhoea-causing superbug bacteria, third-generation cephalosporin-resistant *Escherichia coli*, methicillin-resistant *Staphylococcus aureus*, multidrug-resistant *Pseudomonas aeruginosa*, ciprofloxacin-resistant *Salmonella typhimurium*, and methicillin-susceptible *Staphylococcus aureus*that cause diarrhoea.

## 2. Materials and Methods

### 2.1. Study Design

This was a laboratory experimental study to determine the antibacterial activity of the crude ethanolic and hexane extracts of *Artocarpus heterophyllus* against selected diarrhoea-causing superbug bacteria. The test bacteria were acquired from the Microbiology laboratory at Kampala International University Teaching Hospital. The research was carried out in the Pharmacology and Microbiology laboratories at Kampala International University, western campus. The plant extracts were screened for phytochemicals present and then assayed for the antibacterial activity, Minimum Inhibitory Concentration (MIC) and Minimum Bactericidal Concentration (MBC) using the agar well diffusion and microbroth dilution methods, respectively.

### 2.2. Plant Collection and Materials

The fresh seeds of *Artocarpus heterophyllus* were collected from the Bwegirage village, Ishaka Bushenyi in western Uganda (0.4871 S, 30.2051 E). The seeds were cleaned and the white arils (seed coat) were pulled off and shade-dried for 7 days without removal of the brown spermoderm at room temperature. Dried pieces were finely ground using an electric grinder [23], and the flour obtained was kept at 4°C till further studies.

The materials used included media such as Muller-Hinton agar (HiMedia Laboratories Pvt. Ltd., Mumbai, India, M173) and Brain heart infusion agar for culturing bacteria, positive control antibiotics, ceftriaxone (15 *μ*g) and vancomycin (30 *μ*g), DMSO (10%) as a negative control, and 0.5 McFarland standard solution used as a reference to adjust the turbidity of bacterial suspensions. Equipment such as a gas bath oscillator, dry air oven, aerobic incubator and other apparatus such as Petri dishes, beakers, pipettes, and a ruler among others were used.

### 2.3. Extraction

Extraction was done by maceration of the powdered samples (25 g each) in 100 ml of 80% ethanol and absolute hexane in separate beakers. Extraction was performed with gentle intermittent shaking for five days at room temperature. The extract was filtered using Whatman grade 1 filter paper and concentrated in a steam bath at 45°C. The concentrated extracts were weighed to obtain the extraction efficiency on a dry weight basis. All the remaining extract was stored at 4°C until further use as described by Karthy et al. [23]. The extraction efficiency was calculated as follows: extraction efficiency % = (final dry weight of extract/initial dry weight of plant material) × 100 [24].

### 2.4. Microbial Cultures

The bacterial cultures whose resistance profiles were already established were obtained from the Microbiology laboratory at Kampala International University Teaching Hospital, western campus, for use in the present study and these included third-generation cephalosporin-resistant *Escherichia coli*, methicillin-resistant *Staphylococcus aureus*, multidrug-resistant *P. aeruginosa*, ciprofloxacin-resistant *Salmonella typhimurium*, and methicillin-sensitive *Staphylococcus aureus* ATCC®25923™. Pure cultures of these bacteria were obtained by streak plating on Mueller-Hinton agar and then stored on brain heart infusion agar slants at 4°C till when used [25].

### 2.5. Preparation of Extract Concentration

Using the dried extracts prepared above, two different concentrations of each extract were prepared in mg/ml (500 and 1000) using 10% Dimethyl Sulphur Oxide (10% DMSO) as a diluent since it is a known universal solvent with no antibacterial activity at this concentration [26]. The concentration of 1000 mg/ml was prepared by mixing 2 g of the weighed dried crude extracts in 2 ml of sterile 10% DMSO while 500 mg/ml was made by mixing 1 g of weighed dried crude extract in 2 ml of sterile 10% DMSO contained in a sterile glass beaker. The mixtures were thoroughly dissolved with the help of a spatula and then immediately used in the set experiments. Preliminary trial experiments were performed to establish final working concentrations of 500 mg/ml and 1000 mg/ml for both extracts.

### 2.6. Phytochemical Screening of the Crude Extract

Phytochemical analysis of the seeds crude extract was performed according to the method described by Debiyi and Sofowora [27] and Gul et al. [28]. The phytochemicals screened for included saponins, tannins, flavonoids, alkaloids, cardiac glycosides, reducing sugars, anthraquinones, polyuronides steroids, terpenoids, and amino acids as follows:

  Test for flavonoids: 1.0 ml of 10% lead acetate was added to 1.0 ml of the extract contained in a test tube. The formation of a yellow precipitate was considered positive for flavonoids.
  Test or tannins: 5.0 g of the extract was stirred with 10 ml of distilled water. The mixture was filtered, and the ferric chloride reagent was added to the filtrate. A blue-black precipitate was considered positive for tannins.
  Test for terpenoids: 0.5 ml of the dried extract was evaporated to dryness on a water bath and, then, heated with 3 ml of the concentrated sulphuric acid for ten minutes on a water bath. Formation of a grey colour was an indication of the presence of terpenoids.
  Test for cardiac glycosides: 0.5 g of the dried extract was dissolved in 2.0 ml 0f glacial acetic acid containing a drop of ferric chloride solution. The solution was underlaid with 1.0 ml of concentrated H_2_SO_4_. A brown ring formed at the interface shows the presence of cardenolides.
  Test for saponins: this was screened by shaking 0.5 g of the dried extract with water in a test tube. Frothing which persists on warming was used as evidence for the presence of saponins.
  Test for steroids: 0.5 g of the dried extract was extracted with 2.5 ml of chloroform in a test tube, and 1 ml of concentrated sulphuric acid was added to form a lower layer. A reddish-brown interface was taken to be positive for steroids.
  Testing for reducing sugars: an equal volume of the aqueous extract was added to Fehling's solution followed by boiling in a water bath for 5–10 minutes. The formation of a reddish-brown-coloured (brick red) precipitate due to the formation of cuprous oxide indicated the presence of reducing sugars.
  Test for amino acids: 2.0 g of the extract was dissolved in 10 ml of acetone. A few drops of 2% ninhydrin solution were added to the mixture. The mixture was kept in a water bath for 5 min. A blue or violet colour formed was taken as positive for amino acids.
  Test for anthraquinones: 1.0 g of the ground seed extract was placed in a dry test tube and supplemented with 20 ml of chloroform. This was heated in a steam bath for 5 min. The extract was filtered immediately and allowed to cool. An equal volume of 10% ammonia solution was added to the filtrate. The mixture was shaken, and the upper aqueous layer was observed for pink coloration which was used as indicators for the presence of anthraquinones.

### 2.7. Antibacterial Assay

The antibacterial assay was conducted according to the guidelines set by the clinical and laboratory standards institute [29] with a slight modification. Standardized test bacteria suspensions (equivalent to 0.5 McFarland standard) were inoculated uniformly on the entire surface of freshly prepared Muller-Hinton agar (HiMedia Laboratories Pvt. Ltd., Mumbai, India, M173) using sterile cotton swabs. Wells were made using a sterile cock borer (6 mm). Wells were filled with 50 *μ*L of each concentration (1000 mg/mL and 500 mg/mL) of the crude extracts, and 50 *μ*L of ceftriaxone (15 *μ*g) and vancomycin (30 *μ*g) each were used as positive controls, while 10% DMSO was used as a negative control. The inoculated plates were left at room temperature for 30 min for the extract to diffuse and later, the plates were incubated at 37°C for 24 hours. After the incubation period, the diameter of zones of inhibition were measured in millimetres (mm) using a ruler, and results were interpreted according to guidelines [30]. The experiments were performed in triplicates.

### 2.8. Determination of the Minimum Inhibitory Concentration (MIC)

The minimum inhibitory concentration of the *Artocarpus heterophyllus* ethanolic and hexane crude seed extract was determined for test bacteria that were susceptible using a two-fold serial microbroth dilution method in microtitre plates. Stock concentrations (1000 mg/ml) of ethanol and hexane extracts of *Artocarpus heterophyllus* crude seeds were first prepared by mixing 2 g of weighed dried extract into 2 ml of sterile 10% DMSO contained in a sterile glass beaker. Then, 0.2 ml was picked from this prepared stock (1000 mg/ml) and serially diluted in wells of microtitre plates each containing 0.2 ml of freshly prepared Brain Heart Infusion (BHI) broth to obtain different concentrations ranging from 500, 250, 125, 62.5, 31.25, 15.63, 7.81, 3.91, 1.95, 0.975, 0.49, 0.244, and 0.122 mg/ml. Test bacteria suspension was prepared in 0.85% sterile normal saline, and its turbidity was adjusted to standard 0.5 McFarland equivalent to 1.5 × 10^8^ CFU/ml. This was further diluted by transferring 0.1 ml from this standardized bacteria suspension into a tube containing 9.9 ml of 0.85% sterile normal saline to give a final cell density of 1.0 × 10^6^ CFU/ml which was used in the experiment. The diluted standardized bacterial suspension was added into each of the wells containing the serially diluted crude extract. This was mixed to homogeneity to give a final inoculum of 5 × 10^5^ CFU/ml [31, 32]. Three positive control wells containing broth and test organisms, i.e., methicillin-susceptible *S. aureus*, methicillin-resistant *S. aureus*, and multidrug-resistant P. aeroginosa, were used, while the others containing broth only aimed at checking the ability of the media to support test bacterial growth (bacterial viability) and sterility of broth respectively, the fourth contained broth and crude extract aimed at ascertaining for any prior microbial contamination of the extract. The inoculated microplates were incubated at 37°C for 24 hours. After the incubated period, blanks for each well concentration (extract and BHI only) were prepared, and this was followed by an examination of inoculated wells for visible turbidity by optical density reading at 600 nm with a Beckman DU-70 UV-Vis spectrophotometer. The MIC of the extract was considered as the lowest concentration that had optical density equivalent to its respective blank tube and, thus, had no visible bacterial growth. The test experiments were prepared in triplicates [33].

### 2.9. Determination of the Minimum Bactericidal Concentration (MBC)

Using the MIC microtitre plates, a loop full of the mixture from each of the wells with no visible growth of bacteria after 24 hours of incubation was cultured on freshly prepared Muller-Hinton agar by the streak plate method and was incubated at 37°C for 24 hours. The plates were examined for any colony growth. The least concentration of the extract which had no visible colony growth was considered as the minimum bactericidal concentration [34].

### 2.10. Statistical Analysis

Data generated from the antibacterial effect of the two plant extracts on five test bacteria were entered into Microsoft Excel. These data were then exported to SPSS- version 16 to compute descriptive statistics of the mean and standard error of mean (SEM) inhibition zone diameter. Data were also analyzed with Graph Pad prism 6 to perform one-way analysis of variance (ANOVA) using Tukey's multiple comparisons to compare between different antibacterial activities of different extract concentrations against each test bacteria versus controls. Two-way ANOVA using side's multiple comparison test was used to determine if there were significant differences in the antibacterial activities between the hexane and the ethanolic extracts at varying concentrations against the test bacteria. Statistical significance was considered at *p* ≤ 0.05.

## 3. Results

### 3.1. Percentage Yield of the Extracts

The results of the percentage yield of both the ethanolic and hexane seed extract are shown in Table 1. The results showed that both the ethanolic and the hexane seed extract had 2.2% and 0.9%, respectively.

The results of phytochemical screening in Table 2 showed that both the extracts had flavonoids, tannins, steroids, and reducing sugars. The ethanolic extract had cardiac glycosides and saponins, while hexane extract had anthraquinones.

**Figure 1:**
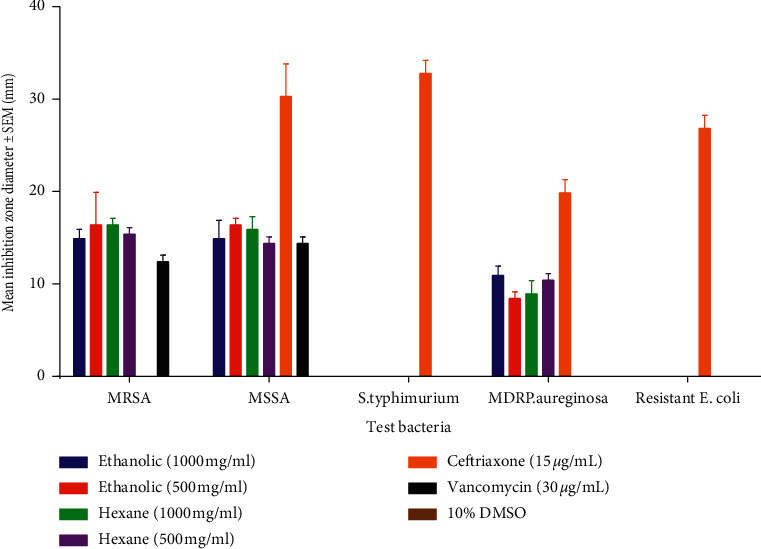
Comparison of mean inhibition zone diameters of the ethanolic and hexane crude extract of *A. heterophyllus* seeds against selected bacteria causing diarrhoea. Key: MRSA: methicillin-resistant *Staphylococcus aureus*, MSSA: methicillin-susceptible *Staphylococcus aureus*, *S. typhimurium*: ciprofloxacin-resistant *Salmonella typhimurium*, MDR PA: multidrug-resistant *Pseudomonas aeruginosa*, R *E. coli*: third-generation cephalosporin-resistant *E. coli*.

**Figure 2:**
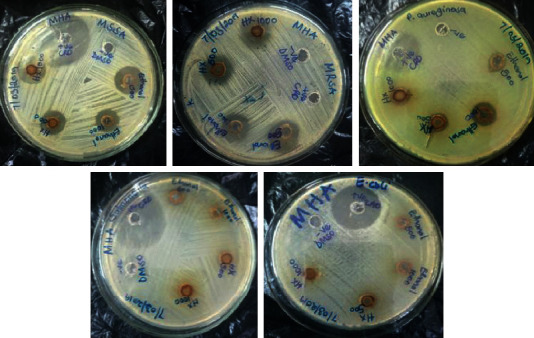
In vitro antibacterial activity of crude extracts of *A. heterophyllus* seeds against selected bacteria using agar well diffusion method.

The results of the antibacterial activity of ethanolic and hexane crude seed extract are shown in Table 3 and Figures 1 and 2. According to the study, both extracts had activity on methicillin-resistant *Staphylococcus aureus*, methicillin-susceptible *Staphylococcus aureus*, and multidrug-resistant *P. aeruginosa*. The extracts did not have activity on third-generation cephalosporin-resistant *E. coli* and ciprofloxacin-resistant *S. typhimurium.*

According to this study, the antibacterial activity of the ethanolic extract at a concentration 500 mg/ml was not significantly different (*p* = 0.8757) from that at 1000 mg/ml and showed inhibition zone diameters of 16.5 ± 2.5 mm and 15.0 ± 1.0 mm, respectively. The ethanolic extract at a concentration of 1000 mg/ml was not significantly different (*p* = 0.8757) from the hexane extract at a concentration of 1000 mg/ml with inhibition zone diameters of 15.0 ± 1.0 mm and 16.5 ± 0.5 mm, respectively. Furthermore, the activity exhibited by the ethanolic extract at a concentration of 500 mg/ml was not significantly different (*p* = 0.9807) from the hexane extract at a concentration of 500 mg/ml. The activity of ethanolic and hexane extracts at a concentration of 1000 mg/ml and 500 mg/ml were significantly different (*p* < 0.0001) from the ceftriaxone (15 *μ*g/ml) which showed no inhibition zone.

The activity of vancomycin (30 *μ*g/ml) on methicillin-resistant *S. aureus* exhibited an inhibition zone diameter of 12.5 ± 0.5 mm which was not significantly different from that of ethanol at 1000 mg/ml (*p* = 0.4039) and hexane at 500 mg/ml (*p* = 0.2080), as shown in Table 3. However, vancomycin had a significantly (*p* = 0.0370) smaller zone diameter of 12.5 ± 0.5 mm compared to ethanol at 500 mg/ml (15.5 ± 0.5 mm) and hexane at 1000 mg/ml (16.5 ± 0.5 mm), as shown in Table 3 and Figure 2.

Considering methicillin-sensitive *Staphylococcus aureus*, the ethanolic extract at 1000 mg/ml was not significantly different (*p* = 0.8757) from the ethanolic extract at a concentration of 500 mg/ml, hexane at 1000 mg/ml (*p* = 0.9807), hexane at 500 mg/ml (*p* = 0.9996), and vancomycin (30 *μ*g/ml) (*p* = 0.6566) which showed an inhibition zone diameters of 15.0 ± 2.0 mm, 16.5 ± 0.5 mm, 16.0 ± 1.0 mm, 14.5 ± 0.5 mm, and 14.5 ± 0.5 mm, respectively. The activity of ethanolic at a concentration of 1000 mg/ml was significantly different (*p* < 0.0001) from that of ceftriaxone (15 *μ*g/ml) and not different from vancomycin antibiotic (30 *μ*g/ml) (*p* = 9996) which had an inhibition zone diameter of 30.5 ± 2.5 mm and 14.5 ± 0.5 mm respectively, and DMSO (10%) (*p* < 0.0001) which had no activity.

However, the activity of ethanolic extract at a concentration of 500 mg/ml was not significantly different (*p* = 0.9996) from that of the hexane extract at a concentration of 500 mg/ml (*p* = 0.6566) with an inhibition zone diameter of 16.0 ± 1.0 mm and 14.5 ± 0.5 mm, respectively (Figure 2). The activity of ethanolicextract at a concentration of 500 mg/ml was significantly different (*p* < 0.0001) from that of ceftriaxone (15 *μ*g/ml) which had a higher inhibition zone diameter of 30.5 ± 2.5 mm and DMSO (10%) (<0.0001) which showed no activity. This was also not significantly different from vancomycin (30 *μ*g/ml) (*p* = 0.6566) with an inhibition zone diameter of 14.5 ± 0.5 mm. Hexane at a concentration of 500 mg/ml was not significantly different (*p* > 0.9999) from vancomycin (30 *μ*g/ml), as shown in Table 3.

In the case of *Salmonella typhimurium*, the activity of the ethanolic extract at a concentration of 1000 mg/ml was not significantly different (*p* > 0.9999) from that of the ethanolic extract at a concentration of 500 mg/ml, hexane at 1000 mg/ml (*p* > 0.9999) and hexane at 500 mg/ml (*p* > 0.9999), and vancomycin (30 *μ*g/ml) (*p* > 0.9999) which had no inhibition zone diameters on the same bacteria. However, the activity of the ethanolic extract at a concentration of 1000 mg/ml was significantly different (*p* < 0.0001) from that of ceftriaxone (15 *μ*g/ml) which showed higher inhibition zone diameters of 33.0 ± 1.0 mm. Still, the activity of the ethanolic extract at a concentration of 500 mg/ml was not significantly different (*p* > 0.999) from that of hexane at 500 mg/ml (*p* > 0.9999), and it exhibited a higher activity compared to vancomycin (30 *μ*g/ml) (*p* > 0.9999) which showed no inhibition zone diameters. The activity of the ethanolic extract at a concentration of 500 mg/ml was significantly lower (*p* < 0.0001) than that of ceftriaxone (15 *μ*g/ml) with an inhibition zone diameter of 33.0 ± 1.0 mm (Figure 2). Furthermore, the activity of hexane at a concentration of 1000 mg/ml was not significantly different (*p* > 0.9999) from that of hexane extract at a concentration of 500 mg/ml, vancomycin (30 *μ*g/ml) (*p* > 0.9999), and DMSO (10%) (*p* > 0.9999) in which the latter had no inhibition zone diameters, as shown in Table 3.

The activity of the ethanolic crude extract on multidrug-resistant *P. aeruginosa* at a concentration of 1000 mg/ml was not significantly different (*p* = 0.4039) from that of ethanolic extract at a concentration of 500 mg/ml, hexane extract at 1000 mg/ml (*p* = 0.6566), and hexane extract at 500 mg/ml (*p* = 9996) with zone diameters of 11.0 ± 1.0 mm, 8.5 ± 0.5, 9.0 ± 1.0 mm, and 10.0 ± 0.5 mm, respectively, and was significantly different from that of ceftriaxone (15 *μ*g/ml) (*p* < 0.0001), vancomycin (30 *μ*g/ml) (*p* < 0.0001), and DMSO (10%) (*p* < 0.0001) which had inhibition zone diameters of 20.0 ± 1.0 mm, 00.0 mm, and 00.0 mm, respectively (Table 3 and Figure 2). The activity of ethanolic extract at a concentration of 500 mg/ml was not significantly different (*p* = 0.9996) from that of hexane extract at a concentration of 1000 mg/ml and that at 500 mg/ml (*p* = 0.6566) with zone diameters of 9.0 ± 1.0 mm and 10.0 ± 0.5 mm, respectively. The activity of ethanolic extract at a concentration of 500 mg/ml was significantly different (*p* < 0.0001) from that of ceftriaxone (15 *μ*g/ml) and DMSO (10%) (*p* < 0.0001) with zone diameters of 20.0 ± 1.0 and 00.0 mm, respectively.

The activity of hexane extract at a concentration of 1000 mg/ml was significantly different (*p* < 0.0001) from that of ceftriaxone (15 *μ*g/ml), vancomycin (30 *μ*g/ml) (*p* < 0.0001), and DMSO (10%) (*p* < 0.0001), as shown in Table 3. The activity of hexane extract at a concentration of 500 mg/ml was significantly different (*p* < 0.0001) from that of ceftriaxone (15 *μ*g/ml) and DMSO (10%) (*p* < 0.0001) that showed no activity. Hexane extract at a concentration of 500 mg/ml was not significantly different (*p* < 0.0001) from vancomycin (30 *μ*g/ml) which had 20.0 ± 1.0 mm and 10.5 ± 0.5 mm zone of inhibition, respectively as shown in Table 3.

The activity of the ethanolic crude extract on third-generation cephalosporin-resistant *Escherichia coli* at a concentration of 1000 mg/ml was not significantly different (*p* > 0.9999) from that at a concentration of 500 mg/ml, hexane extract at a concentration of 1000 mg/ml and 500 mg/ml, DMSO (10%) which showed no zone of inhibition, and vancomycin (30 *μ*g/ml) which showed no activity. The activity of ethanolic extract at a concentration of 1000 mg/ml was significantly different (*p* < 0.0001) from that of ceftriaxone (15 *μ*g/ml) which showed 27.0 ± 1.0 mm as a zone of inhibition (Table 3). Ethanolic extract at a concentration of 500 mg/ml was not significantly different (*p* > 0.9999) from hexane extract at 1000 mg/ml and 500 mg/ml, DMSO (10%), and vancomycin (30 *μ*g/ml) that showed no zone of inhibition. Ethanolic extract at a concentration of 500 mg/ml was significantly different (*p* < 0.0001) from ceftriaxone (15 *μ*g/ml). Hexane extract at a concentration of 1000 mg/ml was not significantly different (*p* > 0.9999) from hexane extract at 500 mg/ml, vancomycin (30 *μ*g/ml), DMSO (10%), and ceftriaxone (15 *μ*g/ml). The hexane extract at a concentration of 1000 mg/ml was significantly different (*p* < 0.0001) from ceftriaxone (15 *μ*g/ml), as shown in Table 3.

The study determined the minimum inhibitory concentration of the *A. heterophyllus* ethanolic and hexane crude seed extract against methicillin-resistant *S. aureus*, methicillin-susceptible *S. aureus*, and multidrug-resistant *P. aeruginosa*, as shown in Table 4. Methicillin-susceptible *S. aureus* had the lowest MIC value of 31.25 mg/ml and 62.25 mg/ml in the ethanolic and hexane crude seed extract of *Artocarpus heterophyllus* seeds, respectively. This was followed by methicillin-resistant *S. aureus* which had MIC values of 62.25 and 125 mg/ml in the ethanolic and hexane crude seed extract of *Artocarpus heterophyllus.* Multidrug-resistant *P. aeruginosa* had the highest MIC values of 125 mg/ml in both the extracts, as shown in Table 4.

### 3.2. Minimum Bactericidal Concentration of Ethanolic and Hexane Crude Extracts of *Artocarpus heterophyllus* Seeds

The study determined the minimum bactericidal concentration of *Artocarpus heterophyllus* crude ethanolic and hexane seed extracts of the test bacteria. Methicillin-resistant *S. aureus* had a higher MBC value of 250 mg/ml for the ethanolic extracts compared to susceptible *S. aureus* whose MBC was at 125 mg/ml. However, both the methicillin-resistant *S. aureus* and methicillin-susceptible *S. aureus* exhibited the same MBC value of 250.0 mg/ml for both extracts. The multidrug-resistant *P*. *aeruginosa* had the highest MBC value greater than 500 mg/ml for both the extracts as shown in Table 5.

## 4. Discussion

It was observed that the percentage yield of ethanolic solvent was higher than that of hexane solvent. This finding was in agreement with reports by Rathi et al. [35] and Deepika et al. [36] who worked on ethanolic plant extracts. This could be attributed to the high solubility of the plant's phytochemicals and other components in ethanol than in hexane. This indicated a high extraction potential of ethanolic solvent.

According to the study, phytochemical analysis of the ethanolic and hexane crude seed extract of *Artocarpus heterophyllus* showed the presence of flavonoids, tannins, steroids, and reducing sugars. The ethanolic crude seed extract had cardiac glycosides and saponins while the hexane extract had anthraquinones. This was in line with the previous study conducted by Sreeletha et al. [37] from India who reported the presence of flavonoids, phenols, phytosterols, carbohydrates, proteins, fats, coumarin, and saponins. Deepika et al. [36] and Moke et al. [20] also reported the presence of saponins, alkaloids, and flavonoids in dichloromethane: ethanol (1 : 1) and acetone crude seed extracts of *A. heterophyllus*. Furthermore, the study showed that terpenoids, amino acids, and anthraquinones were absent in the ethanolic extract while terpenoids, cardiac glycosides, saponins, and amino acids were absent in the hexane extract. Sreeletha et al. [37] reported the absence of flavonoids, saponins, and phenols in the hexane seed extract, and this may explain the lower antibacterial effect exhibited. Studies by Senjobi et al. [22] revealed that the difference in the geographical areas and methods of plant cultivation employed across the world have a great impact on the differences in the phytochemical composition and concentration of the extracts compared to other studies.

*A. heterophyllus* crude seed extracts exhibited antibacterial activity on multidrug-resistant *P. aeruginosa*, methicillin-resistant *S. aureus*, and methicillin-susceptible *S. aureus*. The extracts did not show activity on third-generation cephalosporin-resistant *E. coli* and ciprofloxacin-resistant *S. typhimurium*. Using Tukey's multiple comparison tests, both ethanolic and hexane crude seed extracts at concentrations of 1000 and 500 mg/ml were significantly different from the negative control (10% DMSO) which showed no activity. The variation in the antibacterial activity of the two crude extracts (ethanolic and hexane) could be due to the difference in the bioactive components present in the seed. Still, the antibacterial activity shown by the extracts was in agreement with the study conducted by Binumol and Sajitha [38] who reported that the leaf and bark aqueous extracts from the same plant exhibited antibacterial activity on similar bacteria. Similarly, Sreeetha et al. [37] revealed that the *A. heterophyllus* fruit latex extract had broad-spectrum antimicrobial activity. Their study reported highest zone of inhibition (19 mm) was exhibited by the aqueous extract against *S. aureus*, and the lowest zone of inhibition (7 mm) was observed for the hexane extract against *E. coli*. This antimicrobial activity could be attributed by the bioactive components present in the seeds which included flavonoids, tannins, steroids, reducing sugars, cardiac glycosides, and steroids. These bioactive compounds were reported to have antimicrobial activity [35]. Furthermore, Modilal et al. [39] and Ranasinghe et al. [40] also reported that different researchers [41–43] showed that the presence of these compounds greatly contributes to the antiviral, antibacterial, antihelminthes, anti-inflammatory, and antifungal properties of herbal plants. Moreover, similar studies on plant extracts that contained tannins showed potential antibacterial and antioxidant activities [39, 44, 45]. Furthermore, these findings were in line with studies conducted by Mugweru et al. [46] who reported that flavonoids had antibacterial activity on selected bacteria causing diarrhoea.

Methicillin-susceptible *S. aureus* was more susceptible to both extracts compared to methicillin-resistant *S. aureus* which had a lower zone of inhibition. Multidrug-resistant *P. aeruginosa* was less susceptible to both extracts. Both extracts had activity on all Gram positives while no activity on Gram negatives that were tested. This was in line with Sharma et al. [47], whose study showed that *A. heterophyllus* shell powder had more activity on Gram-positive bacteria (*S. aureus*, *Bacillus subtilis*, *Bacillus cereus*, *Listeria monocytogenes*, and *Streptococcus faecalis*) compared to Gram-negative bacteria (*S. typhimurium*, *Shigella flexneri*, *P. aeruginosa*, and *E. coli*). The authors also showed that *P. aeruginosa* and *E. coli* had lower zones of inhibition. The present study findings were in line with study conducted by Sivagnanasundaram and Karunanayake [48] which revealed that *E. coli* was resistant to leaf extracts of *Artocarpus heterophyllus*. The resistance of Gram-negative bacteria used in this study could be due to the extract's inability to penetrate the bacterial outer membrane. The findings ofthe present study were contrary to the findings reported by Sharma et al. [47] who in their study showed that the *Artocarpus heterophyllus* crude leaf extract had broad-spectrum activity on both Gram-positive and Gram-negative bacteria.

The crude ethanolic extract exhibited the lowest MIC (31.25 mg/ml) required to inhibit methicillin-susceptible *S. aureus* compared to the hexane extract (62.5 mg/ml). The high polarity of ethanol increases its strong interaction with most of the polar phytochemicals, thus having a better extraction potential compared to hexane which extracts mostly nonpolar compounds [49]. A similar finding was revealed by Li et al. [50] who showed that ethanol has a high extraction of polar compounds compared to hexane solvent. Both extracts required a high MIC of 125 mg/ml to inhibit multidrug-resistant *P. aeruginosa*. It can be concluded that Gram-negative bacteria required a higher MIC value in order to cause inhibitory effects as compared to the Gram-positive bacteria. These findings were in agreement with the findings reported by Khan et al. [51].

Furthermore, the present study showed that the crude ethanolic extract required the lowest MBC (125 mg/ml) against methicillin-susceptible *S. aureus* compared to the hexane extract that had the highest MBC (>500 mg/ml) against multidrug-resistant *P. aeruginosa*. The methicillin-susceptible *S. aureus* was more susceptible compared to methicillin-resistant *S. aureus* that had an MBC of 250 mg/ml for both the extracts. The positive findings obtained in this study provide hope for the development of novel antimicrobials with cheap production costs from plants against methicillin-resistant *S. aureus* and multidrug-resistant *P. aeruginosa* as outlined in the sixty-eighth World Health Assembly [18]. This will lower the mortality rates and economic impact associated with these drug-resistant infections in most developing countries [52, 53]. Moreover, several studies have established evidence that the major causes of fatal diarrhoea and bacteria-associated enterocolitis included methicillin-resistant *S. aureus* [54] and multidrug-resistant *P. aeruginosa* [55, 56]; *however, our study findings have shown effectiveness of the* crude seed extracts of *A. heterophyllus against these same superbugs making it a suitable and cheap alternative remedy to be used in treatment*.

## 5. Conclusions

The study showed that the *A. heterophyllus* seed extracts had activity on methicillin-resistant *S. aureus*, methicillin-susceptible *S. aureus*, and multidrug-resistant *P. aeruginosa*while they had no activity on third-generation cephalosporin-resistant *E. coli* and ciprofloxacin-resistant *S. typhimurium*. This could be attributed to the presence of phytochemicals such as flavonoids, tannins, cardiac glycosides, saponins, steroids, reducing sugars, and anthraquinones that were extracted by both ethanolic and hexane solvents. The ethanolic extracts would form better concoctions used in the treatment of infectious diarrhoea caused by methicillin-resistant *S. aureus*, methicillin-susceptible *S. aureus*, and multidrug-resistant *P. aeruginosa*. The study recommends further purification and identification of the different chemical compounds that contribute to the antibacterial activity of *A. heterophyllus* seeds.

## Figures and Tables

**Table 1:** Percentage yield of the *Artocarpus heterophyllus* crude ethanolic and hexane seed extract.

Extracts	Weight of the powder residue (g)	Weight of the extract (g)	Percentage yield (%)
Ethanol	183.75	4.5	2.2
Hexane	333.75	3	0.9

**Table 2:** Phytochemicals present in the ethanolic and hexane crude seed extract of *Artocarpus heterophyllus*.

Phytochemicals	Ethanolic extract	Hexane extract
Flavonoids	+	+
Tannins	+	+
Terpenoids	−	−
Cardiac glycosides	+	−
Saponins	+	+
Steroids	+	+
Reducing sugars	+	+
Amino acids	−	−
Anthraquinone	−	+

Key: +: positive, −: negative.

**Table 3:** Mean and standard error of mean inhibition zone diameters of the ethanolic and hexane crude extract of *A. heterophyllus* seeds against selected bacteria causing diarrhoea.

Mean inhibition zone diameter ± SEM (mm)					
Test bacteria
	MRSA	MSSA	*S. typhimurium*	MDR PA	R *E. coli*
Ethanolic extract					
1000 mg/ml	15.0 ± 1.0^*∗*^	15.0 ± 2.0^*∗*^	0^*∗*^	11.0 ± 1.0^*∗*^	0^*∗*^
500 mg/ml	16.5.25^*∗*^	16.5 ± 0.5^*∗*^	0^*∗*^	8.5 ± 0.5^*∗*^	0^*∗*^

Hexane extract					
1000 mg/ml	16.5 ± 0.5^*∗*^	16.0 ± 1.0^*∗*^	0^*∗*^	9.0 ± 1.0^*∗*^	0^*∗*^
500 mg/ml	15.5 ± 0.5^*∗*^	14.5 ± 0.5^*∗*^	0^*∗*^	10.5 ± 0.5^*∗*^	0^*∗*^

Controls					
Ceftriaxone, 15 *μ*g/mL (+ve)	0^*∗∗*^	30.5 ± 2.5^*∗∗*^	33.0 ± 1.0^*∗∗*^	20.0 ± 1.0^*∗∗*^	27.0 ± 1.0^*∗∗*^
Vancomycin, 30 *μ*g/mL (+ve)	12.5 ± 0.5^*∗*^	14.5 ± 0.5^*∗*^	ND	ND	ND
10% DMSO (−ve)	0^*∗∗*^	0^*∗∗∗*^	0^*∗*^	0^*∗∗∗*^	0^*∗*^

Key: MRSA: methicillin-resistant *Staphylococcus aureus*, MSSA: methicillin-susceptible *Staphylococcus aureus*, *S. typhimurium:* ciprofloxacin-resistant *S. typhimurium*, MDR PA: multidrug-resistant *P. aeruginosa*, R *E. coli*: third-generation cephalosporin-resistant *E. coli*. Different super indexes of an asterisk (^*∗*^, ^*∗∗*^ and ^*∗∗∗*^) in each column of bacteria show a significant difference (*p* < 0.05) within different extract concentration and also between controls, ND: not done.

**Table 4:** Minimum inhibitory concentration (MIC) of the ethanolic and hexane crude extract of *A. heterophyllus* seeds against selected bacteria.

Test bacteria	Minimum inhibitory concentration(mg/ml)	
	Ethanolic extract	Hexane extract
MRSA	62.50	125.00
MSSA	31.25	62.50
MDR PA	125.00	125.00

Key: MRSA: methicillin-resistant *Staphylococcus aureus*, MSSA: methicillin-susceptible *Staphylococcus aureus*, MDR PA: multidrug-resistant *Pseudomonas aeruginosa*.

**Table 5:** Minimum bactericidal concentration (MBC) of the ethanolic and hexane crude extract of *A. heterophyllus* seeds against selected bacteria.

Test bacteria	Minimum bactericidal concentration(mg/ml)	
	Ethanolic extract	Hexane extract
MRSA	250.0	250.0
MSSA	125.0	250.0
MDR PA	>500.0	>500.0

Key: MRSA: methicillin-resistant *Staphylococcus aureus*, MSSA: methicillin-susceptible *Staphylococcus aureus*, MDR PA: multidrug-resistant *Pseudomonas aeruginosa*.

## Data Availability

Data that support the findings of this research are included in the form of tables and figures.
